# Cryo-EM Structure of Heterologous Protein Complex Loaded *Thermotoga Maritima* Encapsulin Capsid

**DOI:** 10.3390/biom10091342

**Published:** 2020-09-19

**Authors:** Xiansong Xiong, Chen Sun, Frank S. Vago, Thomas Klose, Jiankang Zhu, Wen Jiang

**Affiliations:** 1 University of Chinese Academy of Sciences, Beijing 100864, China; xiongxiansong@sibs.ac.cn; 2 Shanghai Center for Plant Stress Biology, Center for Excellence in Molecular Plant Sciences, Chinese Academy of Sciences, Shanghai 201602, China; zhu132@purdue.edu; 3 Department of Biological Sciences, Markey Center for Structural Biology, Purdue University, West Lafayette, IN 47906, USA; sun647@purdue.edu (C.S.); fvago@purdue.edu (F.S.V.); tklose@purdue.edu (T.K.); 4 Department of Horticulture and Landscape Architecture, Purdue University, West Lafayette, IN 47907, USA

**Keywords:** cryo-EM, encapsulin, baculovirus expression system, cargo-loading peptide, complex assembly

## Abstract

Encapsulin is a class of nanocompartments that is unique in bacteria and archaea to confine enzymatic activities and sequester toxic reaction products. Here we present a 2.87 Å resolution cryo-EM structure of *Thermotoga maritima* encapsulin with heterologous protein complex loaded. It is the first successful case of expressing encapsulin and heterologous cargo protein in the insect cell system. Although we failed to reconstruct the cargo protein complex structure due to the signal interference of the capsid shell, we were able to observe some unique features of the cargo-loaded encapsulin shell, for example, an extra density at the fivefold pore that has not been reported before. These results would lead to a more complete understanding of the encapsulin cargo assembly process of *T. maritima*.

## 1. Introduction

Membrane-based organelles in eukaryotic cells are important cellular structures with various functions, including confining enzymes and substrates to enhance enzymatic activity and limit the propagation of potential damage caused by toxic products. In bacteria and archaea, compartmentation is realized by protein-based containers [1,2]. Encapsulin is a kind of protein compartment that exists in a wide variety of bacteria and archaea [3,4,5]. Encapsulins are assembled by capsid proteins into an icosahedral shell with a diameter between 24 and 42 nm, which encapsulates native cargo proteins involved in oxidative stress and iron mineralization [3]. The conserved C-terminal extension of native cargo proteins, which is called cargo-loading peptide (CLP), mediates the interaction between the interior surface of encapsulin and cargo protein [6]. There are also several examples of non-native cargo by fusing the CLP to heterologous proteins like teal fluorescent protein, superfolder green fluorescent protein (sfGFP), and mNeonGreen in *Brevibacterium linens*, *T. maritima*, and *Myxococcus xanthus*, respectively [7,8,9]. However, the conformational changes and the packaging mechanisms of encapsulin and its cargos are poorly understood.

Multiple attempts have been made to solve the structure of encapsulin in *T. maritima* [10], *M. xanthus* [11], and *Pyrococcus furiosus* [3] along with its cargo inside. However, none of these studies were able to obtain a high-resolution cargo structure. In a recent study of the cargo-loaded encapsulins from *Quasibacillus thermotolerans* [12], X-ray crystallography was applied to solve the cargo protein structure before fitting it into the focused refined low-resolution cargo density of the encapsulin map. Considering their programmability and biocompatibility, encapsulin is a promising platform for the delivery of proteins or small ligands and nanoreactors [13]. Using *Escherichia coli* or yeast as a host, nonnative cargo proteins were packaged into encapsulins successfully in vivo [7,8]. However, there are very few studies on the heterologous cargo-loaded encapsulins.

Here, using the insect cell expression system, we encapsulated a heterologous macromolecular protein complex named IDM complex in *T. maritima* encapsulin (hereafter, Encap/IDM complex), which contains Arabidopsis protein IDM1, IDM2, IDM3, HDP1, HDP2, and MBD7 with a total mass of 300 kDa which is the largest cargo so far [14,15], and determined the encapsulin structure using cryo-EM to 2.87 Å resolution. Despite the interference of the inner cargo density, we have successfully solved the encapsulin shell structure with a large cargo inside.

## 2. Materials and Methods

### 2.1. Reconstitution and Purification of IDM Complex-Loaded T. Maritima Encapsulin

The six IDM complex subunit genes were amplified from *Arabidopsis thaliana* cDNA. *T. maritima* encapsulin gene was amplified from pET14-GFP30-Encap (Addgene #86406). The IDM1 C-terminus is tagged with the amino acid sequence of CLP (LFTDKPITEIEEETSGGSENTGGDLGIRKL). All six IDM proteins and C-terminus His-tagged Encap were cloned into pFastBac1 individually. A truncated version of MBD7 was expressed from Ser232 to the end of the C-terminus. For IDM complex expression, a His-sfGFP tag and MBP (maltose-binding protein) tag were fused at the N-terminus of IDM1 and MBD7, respectively. While in Encap/IDM1 and Encap/IDM complex, a C-terminal fused CLP of IDM1 was used instead.

Recombinant baculoviruses were generated in *Spodoptera frugiperda* Sf9 cells (Thermo Fisher Scitific, Waltham, MA, USA). All seven genes were coexpressed in insect cells with a density of 2 × 10^6^ cells per mL and then collected after 60–72 h incubation at 27 °C. Collected cell pellets were stored at −80 °C before protein purification.

The cell pellet was resuspended in buffer A [20 mM HEPES pH 7.5, 150 mM NaCl, 25 mM Imidazole, 0.1 mM Tris (2-carboxyethyl) phosphine hydrochloride (TCEP)] supplemented with Protease Inhibitor Cocktail (TargetMol, Boston MA, USA) and then lysed by sonication. The supernatant was collected after centrifugation at 18,000× *g* for 30 min at 4 °C. The Ni-NTA resin (Cytiva, Marlborough MA, USA) was washed with 10 column volumes of buffer A and then incubated with the supernatant at 4 °C for 1 h. The complex was eluted by buffer B (buffer A with 250 mM Imidazole) after buffer washing and then loaded into Superdex^TM^ 200 10/300 GL column (Cytiva, Marlborough MA, USA) equilibrated with buffer A without imidazole. Fractions were collected and analyzed with SDS-PAGE and coomassie brilliant blue staining.

The reconstitution and purification of encapsulin loaded with IDM1 alone (hereafter, Encap/IDM1) followed the same procedure above with only the baculoviruses of encapsulin capsid and IDM1.

### 2.2. Cryo-EM Sample Grid Preparation

A 4 µL amount of 1 mg/mL of either Encap/IDM or Encap/IDM1 complex sample was applied to glow discharged C-flatTM CF-2/1-4Cu-50 grids (Electron Microscopy Sciences, Hatfield PA, USA) and plunge frozen with a Cp3 plunger (Gatan, Inc., Pleasanton CA, USA), 90% humidity, ashless filter paper (Whatman^®^ #1001-032)(Cytiva, Marlborough MA, USA), and 2 s of blotting.

### 2.3. Cryo-EM Data Acquisition

Images of Encap/IDM1 were taken on Talos F200C transmission electron microscope (Thermo Fisher Scientific, Waltham, MA, USA) recorded by the Ceta 16M camera (Thermo Fisher Scientific, MA, USA) with a pixel size of 2.04 Å.

The sample grid of Encap/IDM complex was imaged with a Titan Krios transmission electron microscope (Thermo Fisher Scientific, MA, USA). Images were recorded using a Gatan K3 Summit detector (Gatan, Inc., Pleasanton CA, USA) mounted on a Gatan Quantum energy filter (Gatan, Inc., Pleasanton CA, USA) using a 20 eV zero-loss slit in super-resolution counting mode. The movies were collected using Leginon software [16] at a dose-rate of 30 electron/pixel/second. A nominal magnification of 81,000 was used for data collection resulting in 0.536 Å super-resolution pixel size. The total dose of each movie was 56.4 electrons/Å2 from 2.16 s exposure. Detailed imaging conditions for the dataset are listed in Table 1.

### 2.4. Image Processing

The dataset of Encap/IDM complex consisted of 2175 movies. The raw movies were aligned and dose-weighted with Motioncor2/1.0.5 [17]. The motion-corrected micrographs were imported into cryoSPARC/v2 [18] for the following processing steps. A total of 1,535,389 particles were extracted by template matching particle picking. After multiple rounds of heterogeneous refinement, a homogenous set of 112,241 particles was used in ab initio reconstruction with C1 symmetry and the final homogeneous refinement with icosahedral symmetry in cryoSPARC/v2 to 3.39 Å resolution. Further icosahedral refinement with JSPR [19,20] resulted in 2.87 Å resolution based on the “gold standard” Fourier Shell Correlation at FSC = 0.143 criterion. The same set of particles was also used for homogenous refinement with C1 symmetry to 4.29 Å resolution in cryoSPARC/v2. Detailed processing statistics for the dataset are listed in Table 1. The electron density map of the encapsulin shell was deposited in the Electron Microscopy Data Bank (EMDB; https://www.ebi.ac.uk/pbde/emdb) under accession number EMD-22617.

### 2.5. Model Refinement

The PDB model 3DKT was fitted into the electron density maps in Chimera [21] and refined with Rosetta [22,23]. The refined monomer was visualized in Coot [24,25], refined with PHENIX [26], and then subjected to symmetry refinement in a pentamer unit with Rosetta symmetry refinement. The refined atomic model has been deposited in the Protein Data Bank (PDB; https://www.rscb.org/) under accession number 7K5W.

## 3. Results

### 3.1. Self-Assembly of Heterologous Macromolecular Cargo-Loaded Encapsulin in Baculovirus Expression System

**Figure 1:**
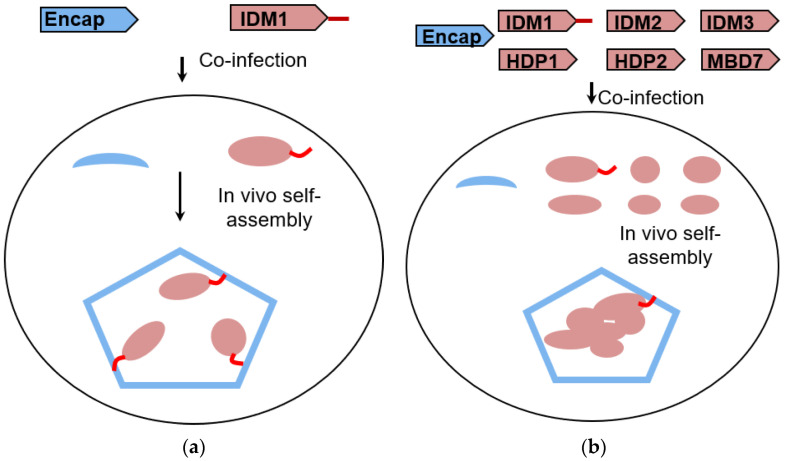
Schematic representation of heterologous cargo-loaded encapsulin expression in insect cells. Diagrams of in vivo encapsulin assembly resulted from the coexpression of Encap and cargo proteins in the baculovirus expression system with IDM1 as cargo alone (**a**) and the IDM holo complex as cargo (**b**).

**Figure 2:**
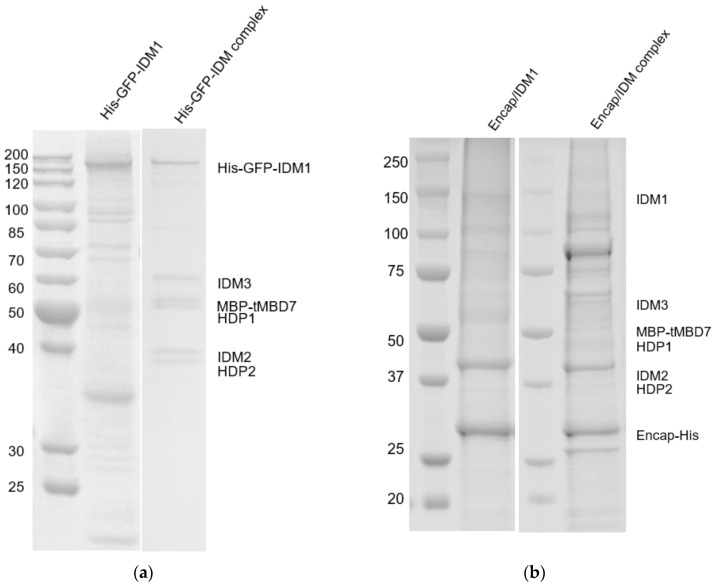

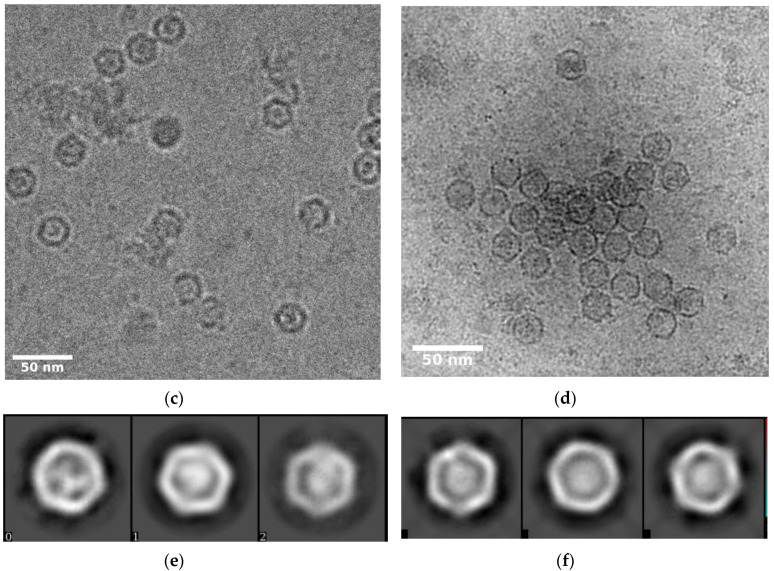
Production of heterologous cargo-loaded encapsulin. The SDS-PAGE result of His-GFP-IDM1 and His-GFP-IDM complex (**a**), Encap/IDM1 and Encap/IDM complex (**b**). His-GFP-IDM1: His-GFP fused at the N-terminal of IDM1. The IDM complex without encapsulin (**a**) was purified with the His-tag on the N terminal of IDM1 protein. In the Encap/IDM complex, there was no His-tag on IDM1 but was at the C-terminal of encapsulin capsid (Encap). The molecular weight of His-GFP tag, MBP tag, IDM1, IDM2, IDM3, HDP1, HDP2, and MBD7 are 32kDa, 40kDa, 131kDa, 39kDa, 52kDa, 46kDa, 33kDa, and 35kDa, respectively. Cryo-EM image of Encap/IDM1 (**c**) from Talos F200X G2 and Encap/IDM complex (**d**) from Titan Krios. Representative 2D class averages generated from the Encap/IDM1 (**e**) and the Encap/IDM complex dataset (**f**). The “matchto” processor in EMAN2 [27] accessed through *e2proc2d.py* was used to filter the 2D class averages to the same level by matching their structure factor curves.

To encapsulate the IDM complex in vivo, six subunits of IDM and encapsulin from *T. maritima* were constructed into individual operons and coexpressed in the insect cells. Based on cross-linking coupled mass spectrometry analysis (data not shown), the C-terminal end of IDM1 (the largest subunit of the IDM complex) was free from protein–protein interactions within the IDM complex. The 30 amino acid cargo-loading peptide was fused at the C-terminal of the IDM1 protein (Figure 1a,b). Purification of the encapsulin particles was accomplished through Ni-NTA affinity of the surface-exposed His-tag at the C-terminal of the encapsulin protein, followed by gel filtration. The confirmation of the copurification of encapsulin and the IDM complex was done by SDS-PAGE (Figure 2a,b). As a pilot test, we first only incorporated the IDM1 protein alone into the encapsulin and observed the additional density inside of the encapsulin shell as shown in Figure 2c. Encouraged by the successful packaging of the IDM1 protein, we then tried to incorporate the whole IDM complex, hoping that the whole IDM complex could be packaged inside of encapsulin. With IDM1 alone containing the cargo-loading peptide, the IDM complex should have been preassembled before being loaded into encapsulin. The cryo-EM image of purified particles of the whole IDM complex (Figure 2d) indeed had larger density inside the shell compared to the IDM1 protein alone loaded encapsulin (Figure 2c) as also shown in the 2D class averages of the Encap/IDM1 (Figure 2e) and Encap/IDM complex (Figure 2f). It indicates a successful assembly of the heterologous IDM complex inside of the encapsulin shell with the insect cell expression system. However, we did notice a significant number of contaminant bands in the SDS-PAGE gel. We reasoned that since only the encapsulin capsid had the His-tag, if we could see the bands of both the proteins, they must have been copurified as a complex.

### 3.2. Overall Structure of IDM Complex-Loaded T. maritima Encapsulin

**Figure 3:**
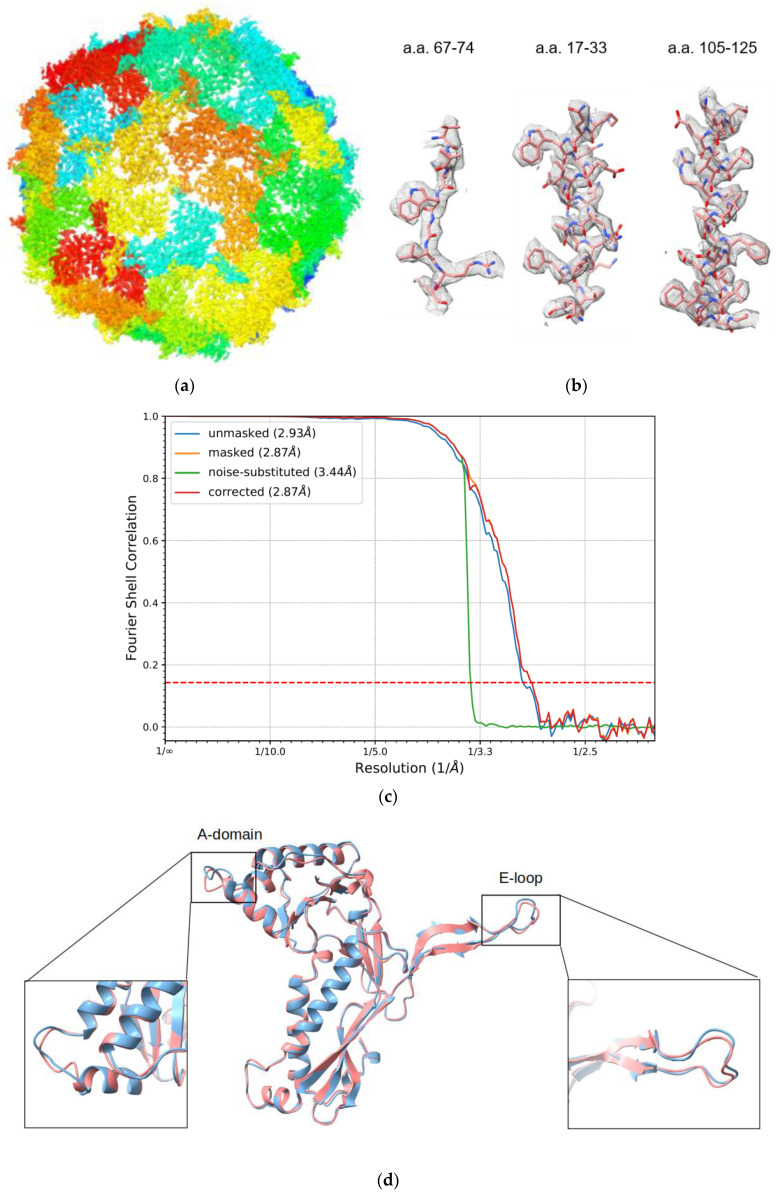
Overall structure of IDM complex-loaded *T. maritima* encapsulin. (**a**) The sharpened density map of Encap/IDM complex from the icosahedral reconstruction. (**b**) The closeup view of the side-chain densities of short segments of a.a.67-74, a.a.17-33, and a.a.105-125. (**c**) The FSC curve of the reconstructed cryo-EM half maps. (**d**) Structure alignment of the Encap (coral) and Encap/IDM monomer (light blue).

Using single-particle cryo-EM, we determined the structure of *T. maritima* encapsulin packaged with a heterologous IDM complex (Figure 3a). The overall resolution of the encapsulin icosahedral shell was solved at 2.87 Å based on the gold standard Fourier Shell Correlation (FSC) 0.143 threshold (Figure 3c). To evaluate the cryo-EM density map, the crystallographic model of *T. maritima* encapsulin (PDB:3DKT) [6] was docked into our density map. The crystal structure was further refined into the 2.87 Å resolution cryo-EM density map. The refined model fit the density map very well including the sidechain densities (Figure 3b). After superimposing the refined model and original crystal structure of encapsulin (PDB:3DKT), the two structures were largely the same (RMSD = 0.681 Å) with only minor differences at the A-domain and the E-loop (Figure 3d). Both the A-domain loops and E-loops were slightly shifted without changing the overall shell structure of the Encap/IDM complex. It is consistent with the idea that A-domain loops and E-loops are the most flexible regions of encapsulin capsid protein. The electron density fitted monomer model demonstrating the map quality at E-loop and A-domain are presented in Figure S1. We did notice that there were missing densities at the Gly189 and Ala188 position, which was probably due to the flexibility of the A-loop. The density of those two residues were also missing the PDB-3DKT X-ray density map.

### 3.3. Pores of the Encap/IDM Complex

**Figure 4:**
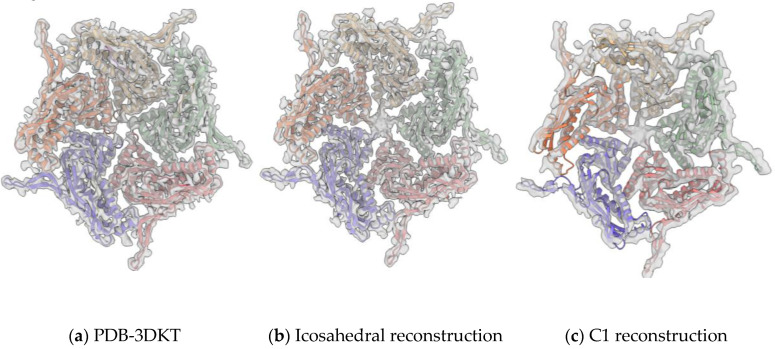
Pores of the heterologous IDM-loaded encapsulin. The inner surface view along the fivefold axis of Encap/IDM complex. From left to right: the X-ray density map of PDB-3DKT (**a**), the crystal model of *T. maritima* fitted into the icosahedral reconstructed density map of our dataset (**b**), and the C1 symmetry reconstructed map (**c**).

**Figure 5:**
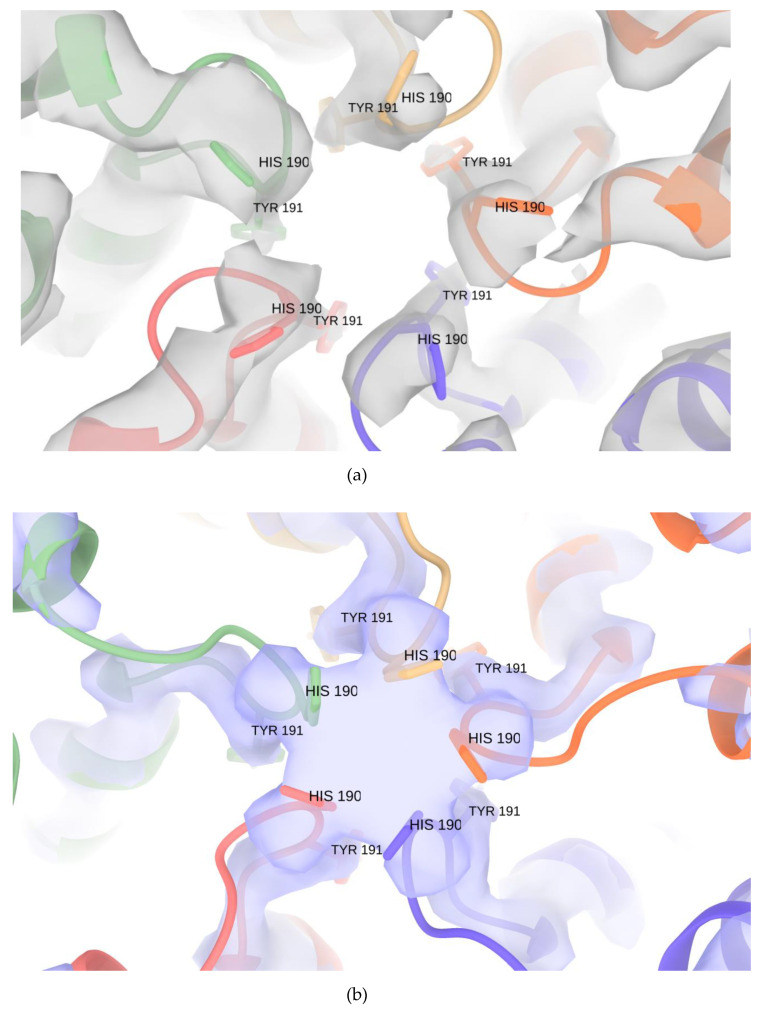
Conformation comparison of the crystal encapsulin structure (PDB:3DKT) fitted in the X-ray 2fo-fc map (grey) (**a**) and Encap/IDM complex (**b**) refined model fitted in the EM density map (purple) at the fivefold pore.

The crystal structure of *T. maritima* encapsulin has multiple openings in its capsid shell, including the positively charged threefold pore and uncharged fivefold pore (Figure 4a) [6]. The openings were thought to be the channel for the substrates and/or products of the cargo protein. In our IDM-loaded encapsulin structure, we have found extra density at the fivefold pore in both icosahedral shells (Figure 4b). To test if the extra densities were an image-processing artifact of icosahedral reconstruction along the symmetric axes, we then performed de novo C1 reconstruction and found extra densities at all 12 fivefold pores in the C1 reconstruction (Figure 4c). The extra density should thus be authentic structural features of the IDM-loaded encapsulin that might be caused by substrate binding or cell factors copurified from the insect cell. The C1 reconstructed map was refined to 4.29 Å resolution. The cryoSPARC-reported FSC curve is shown in Figure S3. Some part of the C1 reconstructed map is less resolved compared to the icosahedral map. We reasoned that when we performed the C1 symmetry refinement, the orientation alignment of the icosahedral shell of encapsulin was affected more by the cargo density. When we applied icosahedral symmetry, alignment was less vulnerable to the cargo density which did not have icosahedral symmetry and would have been averaged out in the icosahedral reconstruction. A zoom-in view of the extra density at the fivefold pore and conformational comparison of Encap and Encap/IDM complex is presented in Figure 5.

### 3.4. Structure of Encapsulated IDM Complex

We designed this experiment with the intention to protect the IDM complex from the air–water interface with the encapsulin shell and hoped to get a more uniform state of the IDM complex with the confined space inside of the shell. We have tried multiple image-processing software such as RELION and cryoSPARC and strategies including computational subtraction of the encapsulin shell signals to reconstruct the structure of the IDM complex inside of the Encap/IDM complex. However, we failed to obtain a good model for the IDM complex. It could be due to the heterogeneous compositions/conformations, low signal-to-noise ratio of the images, and interferences from the signals of the shell. In view of this failure, an attempt was made to recover the density of the CLP near the threefold axis with symmetry expansion and focused refinement in RELION. However, after multiple trials, the 3D classes of focused classification only contained the shell density. The density of CLP was expected to be hard to resolve since there was only one CLP per capsid, while when we did symmetry expansion, it would have been overwhelmed with the other expanded 59 sets of particles without the CLP. Therefore, even with a small number of particles having the loading peptide density, it could still be averaged out during the reconstruction. Although not being able to recover the CLP density at the threefold pore, we observed strong protein density inside of the encapsulin capsid in the focused refined map (Figure S2), which is another evidence of the existence of the inner protein complex. Future optimization of sample quality will be needed, for example, multiple CLPs fused to additional IDM subunits to enhance binding affinity and specificity, to allow successful structural determination of the encapsulated IDM complex.

## 4. Discussion

Our study is distinct from previous studies in three aspects. Firstly, we are presenting the first case of insect cell expression of encapsulin. As a reprogrammable platform, encapsulin has been expressed in several hosts such as *E. coli*, yeast, and mammalian cells but not insect cell systems. This project was designed to use encapsulin as a platform for solving protein complex structures that are particularly vulnerable to the air–water interface problem. The insect cell system has an advantage in expression of many eukaryotic proteins that require post-translational modifications and cannot be expressed in bacterial systems. This demonstration of successful expression and formation of an intact encapsulin shell expands potential cargo targets that require insect cell expression systems.

Secondly, we have demonstrated the capability of coexpressing encapsulin and a heterologous cargo in the insect cell expression system. In a previous crystallography study, the *T. maritima* encapsulin was purified directly from its bacterial host and some of them might have contained native cargo [6]. In our study, encapsulin and cargo proteins were cloned separately in seven operons and self-assembled in vivo. The density inside of encapsulin in the TEM images indicated successful cargo loading and assembly.

Thirdly, the cargo that we chose to assemble inside of encapsulin was not a single protein but a multi-subunit complex which contributed to stronger cargo density inside of the encapsulin shell. Despite this, we were able to obtain a 2.87 Å encapsulin shell structure with a dataset of Encap/IDM complex. It is not surprising that we could not resolve the inner cargo structure and the loading peptide density, probably due to sample quality, the intrinsic flexibility of the IDM complex, the limited number of particles, and the interference of the shell signal. There is no doubt that the structure of the IDM complex inside and the loading peptide density on the shell would be more interesting findings. However, to characterize these further and hence to be able to shed light on the encapsulin assembly conformational changes, it will require significant improvement in both sample preparation and image-processing technique. Thus, if we can better handle these problems, it should be possible in the future to obtain an interpretable map of the IDM complex inside of encapsulin.

## Figures and Tables

**Table 1:** Statistics.

Data Collection	Value
Voltage (kV)	300
Total dose (e^−^/Å2)	56.4
Nominal magnification	81,000
Super-resolution pixel size (Å)	0.536
Number of movies	2175
Number of frames/movies	40
Intended defocus (μm)	0.5–2
**Image processing**	
Particles picked	1,535,389
Final number of particles	112,241
Refined resolution (Å)	2.87
**Refinement**	
Map CC	0.8576
All-atom clashscore	5
Rotamer outliers (%)	0
**Ramachandran plot**	
Favored (%)	96.56
Allowed (%)	3.44
Outliers (%)	0

## Supplementary Materials

The following are available online at https://www.mdpi.com/2218-273X/10/9/1342/s1, Figure S1: The Rosetta refined monomer model superimposed with the density map, Figure S2: The extra density of the inner capsid in the focused refinement of the threefold pore, Figure S3: The FSC curve of C1 symmetry reconstructed map reported by cryoSPARC.
