# Supplementary material for: Cryo-EM Structure of Heterologous Protein Complex Loaded Thermotoga Maritima Encapsulin Capsid
Source: Biomolecules. 2020 Sep 19;10(9):1342. doi: 10.3390/biom10091342 (PMC7565527; doi:10.3390/biom10091342)
Supplement: Supplementary file 1 [file biomolecules-10-01342-s001.pdf]

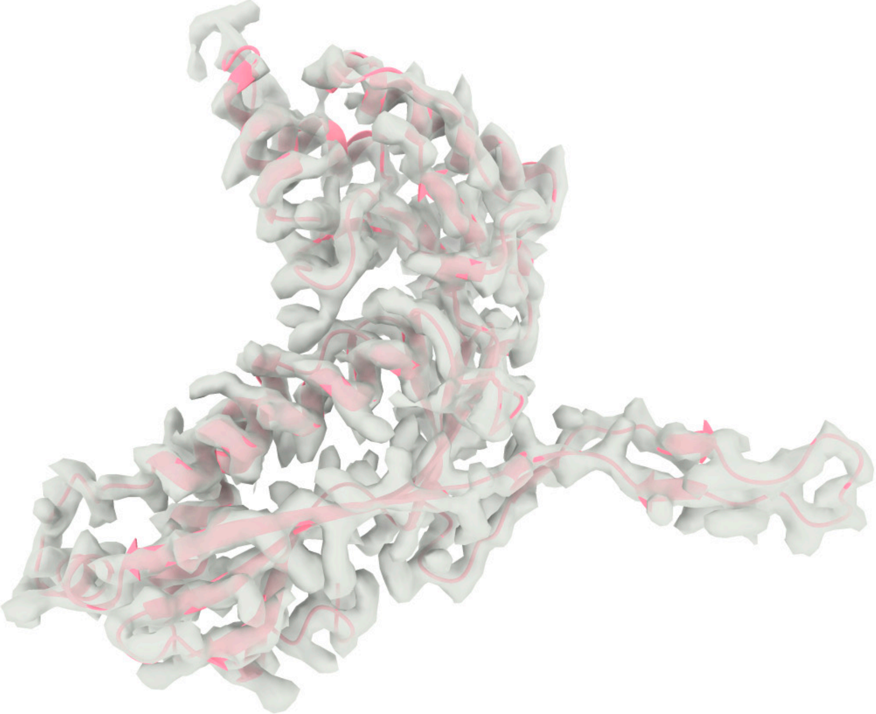

(a)

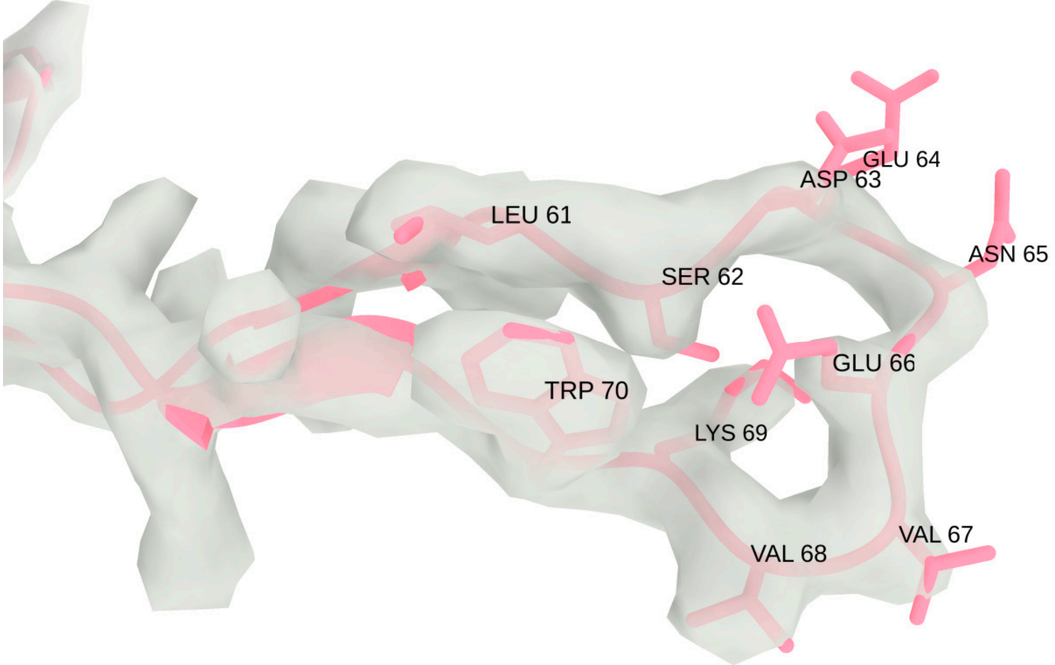
GLU 64
ASP 63
LEU 61
ASN 65
SER 62
GLU 66
TRP 70
LYS 69
VAL 67
VAL 68

(b)

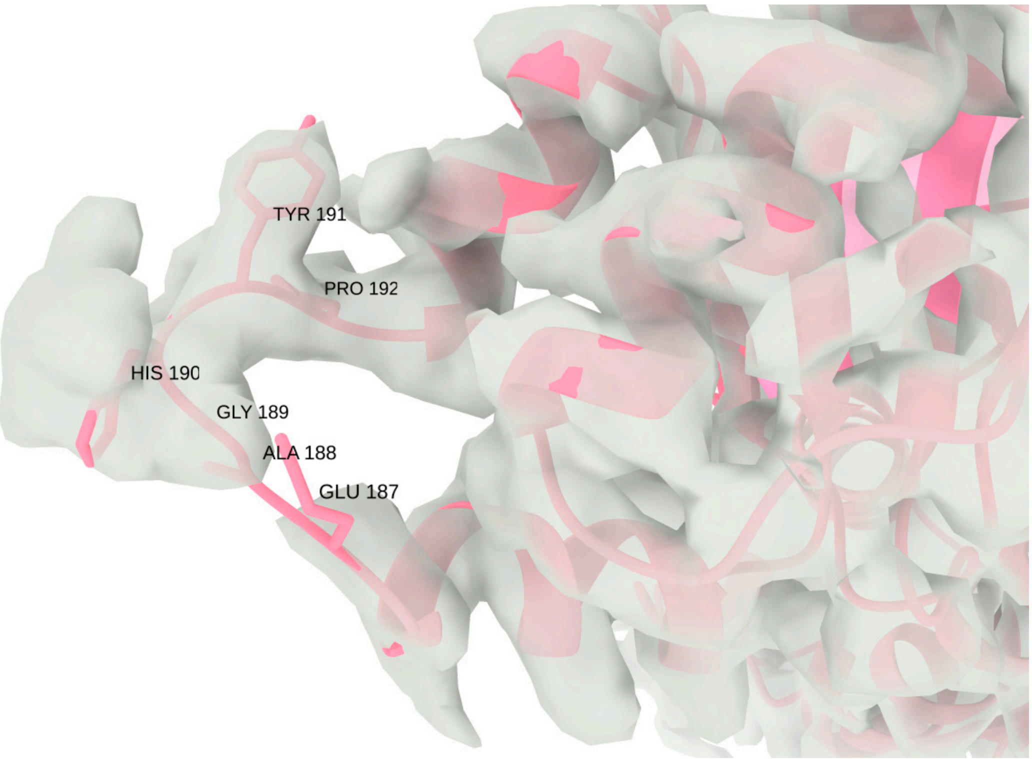

(c)

Figure S1. The ROSETTA refined monomer model (coral) fitted into the density map (grey) (a). The map was sharpened by *phenix.auto_sharpen*. The zoom-in view of the E loop (b) and A-domain (c) with key residues shown in stick. The side chain densities of Trp70, Leu61 and Val68 are well resolved in our density map. Although the side chain densities of the other residues were missing or partially missing, the density map is able to provide enough information to model the backbone of the E loop.

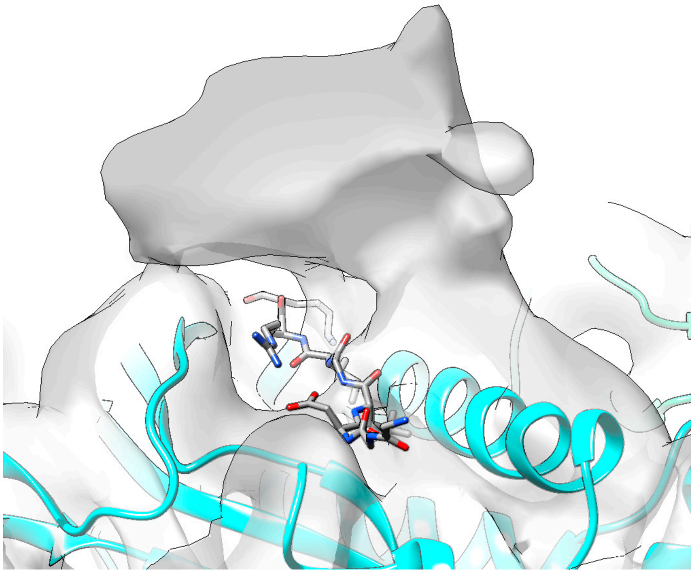

Figure S2. The extra density of the inner capsid in the focused refinement of the three-fold pore region. Although there is no density for the putative cargo loading peptide (shown in stick), we can see the strong protein density connected to the inner surface of the encapsulin shell.

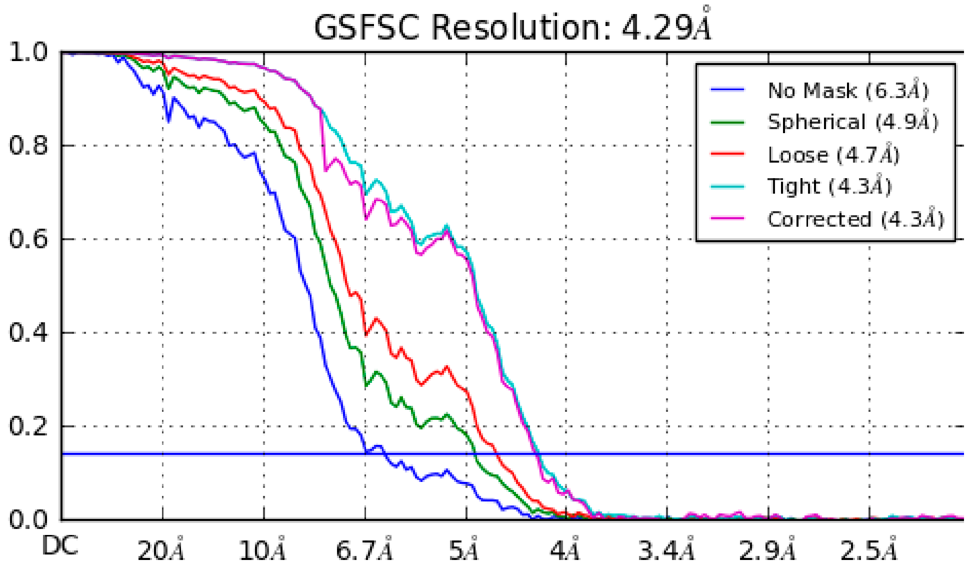

Figure S3. The FSC curve of C1 symmetry reconstructed map reported by cryoSPARC.
